# Effect of surgical liver resection on circulating tumor cells in patients with hepatocellular carcinoma

**DOI:** 10.1186/s12885-018-4744-4

**Published:** 2018-08-20

**Authors:** Jing-jing Yu, Wei Xiao, Shui-lin Dong, Hui-fang Liang, Zhi-wei Zhang, Bi-xiang Zhang, Zhi-yong Huang, Yi-fa Chen, Wan-guang Zhang, Hong-ping Luo, Qian Chen, Xiao-ping Chen

**Affiliations:** 1 Translational Medicine Center, Tongji Hospital, Tongji Medical College, Huazhong University of Science and Technology, Wuhan, 430030 People’s Republic of China; 2 Hepatic Surgery Center, Tongji Hospital, Tongji Medical College, Huazhong University of Science and Technology, Wuhan, 430030 China; 3 Division of Gastroenterology, Department of Internal Medicine, Tongji Hospital, Tongji Medical College, Huazhong University of Science and Technology, Wuhan, 430030 China

**Keywords:** Circulating tumor cells, Perioperative period, Hepatocellular carcinoma, Liver resection, Disease-free survival, Overall survival

## Abstract

**Background:**

This study explored the effect of liver resection on perioperative circulating tumor cells (CTCs) and found that the prognostic significance of surgery was associated with changes in CTC counts in patients with hepatocellular carcinoma (HCC).

**Methods:**

One hundred thirty-nine patients with HCC were consecutively enrolled. The time-points for collecting blood were one day before operation and three days after operation. CTCs in the peripheral blood were detected by the CellSearch™ System.

**Results:**

Both CTC detection incidence and mean CTC counts showed greater increases postoperatively (54%, mean 1.54 cells) than preoperatively (43%, mean 1.13 cells). The postoperative CTC counts increased in 41.7% of patients, decreased in 25.2% of patients and did not change in 33.1% of patients. The increase in postoperative CTC counts was significantly associated with the macroscopic tumor thrombus status. Patients with increased postoperative CTC counts (from preoperative CTC < 2 to postoperative CTC ≥ 2) had significantly shorter disease-free survival (DFS) and overall survival (OS) than did patients with persistent CTC < 2. Patients with persistent CTC levels of ≥2 had the worst prognoses.

**Conclusions:**

Surgical liver resection is associated with an increase in CTC counts, and increased postoperative CTC numbers are associated with a worse prognosis in patients with HCC.

**Electronic supplementary material:**

The online version of this article (10.1186/s12885-018-4744-4) contains supplementary material, which is available to authorized users.

## Background

Hepatocellular carcinoma (HCC) accounts for 90% of primary liver cancers and is the second most common cause of cancer-related deaths worldwide [1]. Currently, surgery is the first choice of treatment for this disease. Resection and liver transplantation achieve excellent results in early-stage patients [2], however, recurrence and metastasis are frequently seen post-resection, and approximately 40% of patients develop recurrences within the first year after hepatectomy [3]. Therefore, it is imperative to address those factors in the perioperative period that foster the capture and promotion of metastases to control residual malignant cells and improve long-term oncological outcomes.

Recent evidence has demonstrated that surgery, which is intended to be a curative option for removing and reducing the tumor mass to eliminate the cancer may increase the establishment of new metastases and accelerate growth of residual and micro-metastatic disease by generating a permissive environment for metastasis. This includes increased shedding of cancer cells into the bloodstream and suppressing antitumor immunity, thus allowing tumor cells to survive in the circulation [4–6]. However, whether surgical procedures introduce additional circulating tumor cells (CTCs) into the bloodstream remains controversial, as other studies have shown that CTC counts normalize and often decrease after surgery [7, 8]. More importantly, the long-term effects that surgically released CTCs have on progression and survival remain unknown [9]. Several reports have demonstrated that increased postoperative CTC numbers were associated with worse prognoses in lung and colon cancers [10, 11], while one study on pancreatic cancer found no such relationship [12]. Therefore, diverse surgical operations for different solid cancers should be individually investigated, as the specific protocols of surgical tumor manipulation may be critical and may influence the outcomes.

Few data are available for evaluating possible modifications of CTC detection in the perioperative period of patients undergoing surgery for operable HCC. This study explored the effect of liver resection on perioperative CTCs and found that the prognostic significance of the surgery caused changes in CTC counts in patients with HCC. This information may increase our knowledge of the biology of the metastatic process, and particularly of the impact of surgery on the release of cells into the bloodstream.

## Methods

### Patients

One hundred thirty-nine patients with HCC and 23 control patients with benign hepatic tumors (cavernous hemangioma) were consecutively enrolled between December 2013 and June 2015 at the Hepatic Surgery Center, Tongji Hospital, Tongji Medical College, Huazhong University of Science and Technology. The inclusion criteria were (1) definitive pathological diagnosis of primary HCC; (2) received curative resection, defined as complete macroscopic tumor removal; (3) margin-negative R0 resection; (4) no ablation used at the time of resection; (5) no prior anticancer treatment; and (6) aged between 18 and 80 years. Exclusion criteria were (1) with distant metastasis and (2) having other active or preexisting malignancies. All surgical procedures were performed in this department, and the same surgical and oncological principles were followed. The institutional review board approved the study protocol, and all patients provided written informed consent.

### CTC analysis

Preoperative peripheral blood specimens were collected one day before surgery. To determine the postoperative time-point for blood collection, CTCs were detected in peripheral blood specimens collected immediately after surgery, three days after surgery and seven days after surgery in 12 HCC patients (Additional file 1). Because the postoperative CTC counts showed no significant differences between the three time-points (Wilcoxon matched-paired signed rank test, *P* > 0.05), three days after surgery was used as the postoperative time-point for collecting blood.

Briefly, peripheral blood specimens (7.5 mL) were drawn into CellSave Preservative Tubes (Janssen Diagnostics, LLC, Raritan, NJ, USA), stored at room temperature and processed within 96 h after collection. To avoid possible contamination with epithelial skin cells, one extra tube (5 mL) for other detections was filled before the assay tube. The CellSearch™ System was used for detecting and counting CTCs as previously described [13]. Briefly, tumor cells were immunomagnetically captured away from the peripheral blood cells using iron beads coated with anti-EpCAM monoclonal antibody (mAb) and then identified by fluorescence microscopy using the following definitions: cytokeratin-positive, CD45-negative, and nucleated.

### Statistical analysis

Patients were followed until April 15, 2016. To be certain all deceased patients were counted, we reviewed the governmental death registration and made telephone follow-ups. Disease-free survival (DFS) and overall survival (OS) were estimated by Kaplan-Meier analysis and compared using the log-rank test. A Cox proportional hazards model was used to identify factors associated with DFS and OS, and those factors at *P* < 0.05 in the univariate analysis were included in the multivariate models. A chi-squared test and Fisher’s exact test were used for between-group comparisons as appropriate. *P* < 0.05 was considered statistically significant. All statistical analyses were performed using SPSS version 21.0 for Windows (IBM).

## Results

### Patient characteristics

Table 1 summarizes the clinical demographics and tumor characteristics of the 139 patients with HCC enrolled in our study. The mean (±SD) age of the patients was 49.9 ± 10.3 years (range 24–77 years), and 87.8% were male. Of these patients, 84.9% were hepatitis B surface antigen (HBsAg)-positive, and two were also positive for the hepatitis C virus (HCV). Of these patients, 74.1% had liver cirrhosis, and 71.9% were α-fetoprotein (AFP)-positive. Most patients (95.0%) had normal hepatic function (Child-Pugh score A), and 7 who were classified as Child-Pugh score B received short-term liver protective therapy before surgery. Tumor stage was determined per the Barcelona Clinic Liver Cancer (BCLC) staging system. The proportion of stage 0 + A was 40.3%.

**Table 1:** Clinical characteristics of 139 HCC patients

Clinical characteristics	No. of patients
Preoperative	
Age, years	Mean: 49.9 ± 10.3; Median: 48.0
Sex	
Male	122
Female	17
HBsAg	
Negative	21
Positive	118
Liver cirrhosis	
No	36
Yes	103
Child-Pugh score	
A	132
B	7
Operative	
Operation method	
Open	123
Laparoscopic	16
Operation time (min)	Mean: 245.94 ± 83.22; Median: 236.00
Blood loss (ml)	Mean: 447.12 ± 636.25; Median: 200.00
Blood transfusion	
Yes	28
No	111
Hepatic vascular occlusion	
Yes	83
No	56
Tumor characteristics	
Largest tumor size, cm	
≤ 5	61
> 5	78
No. of tumors	
Single	106
Multiple	33
Macroscopic tumor thrombus	
No	113
Yes	26
Vascular invasion	
No	84
Yes	55
BCLC stage	
0 + A	56
B + C	83
AFP, ng/mL	
Negative (≤ 7.0)	39
Positive (> 7.0)	100

### Preoperative and postoperative CTC counts

**Fig. 1:**
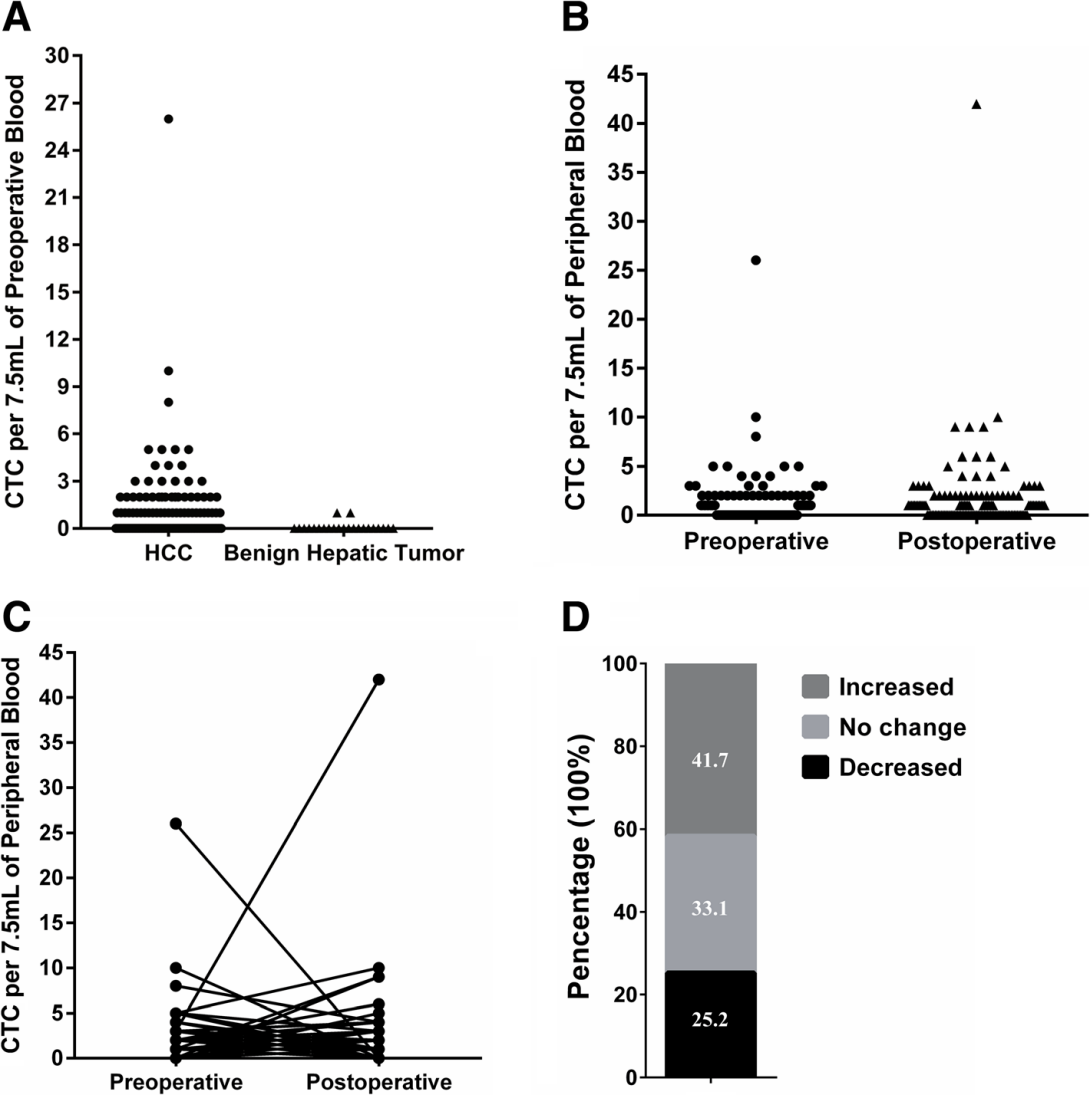
Comparison of perioperative CTC counts in patients with HCC and benign hepatic tumors. **a** Frequency distribution of preoperative CTC counts in HCC and benign hepatic tumor patients; **b** Frequency distribution of preoperative and postoperative CTC counts in 139 HCC patients; **c** Ladder plots displaying preoperative and postoperative CTC counts for each HCC patient; **d** Incidence of increase, decrease or no change in the postoperative CTC counts relative to the preoperative CTC counts from the same HCC patient

A comparison of the preoperative CTC counts for both the HCC and benign hepatic tumor patients is shown in Fig. 1a. Two of the 23 patients with benign hepatic tumors had 1 CTC; the remaining patients had 0. The frequency distribution of preoperative and postoperative CTC counts in HCC patients was shown in Fig. 1b. The preoperative and postoperative CTC detection incidences were 43.9% and 54.0%, respectively. The mean CTC counts also increased postoperatively (mean 1.54 cells, range 0–42 cells) versus preoperatively (mean 1.13 cells, range 0–26 cells), but the difference was not statistically significant (Wilcoxon matched-paired signed rank test, *P* = 0.1158). Ladder plots displayed preoperative and postoperative CTC counts for each of the 139 HCC patients (Fig. 1c). Compared with the preoperative CTC counts, the postoperative CTC counts increased in 58 (41.7%) patients, decreased in 35 (25.2%) patients and did not change in 46 (33.1%) patients (Fig. 1d).

The association between the change in perioperative CTC counts and HCC patient characteristics was analyzed. As shown in Table 2, the increase in postoperative CTC counts was significantly associated with the macroscopic tumor thrombus condition: CTCs increased postoperatively in 17/26 (65.4%) patients with macroscopic tumor thrombus versus in 41/113 (36.3%) patients without macroscopic tumor thrombus (*P* = 0.012). Postoperative CTC count changes were not significantly associated with age, sex, hepatitis B viral (HBV) infection, liver cirrhosis, Child-Pugh score, AFP, tumor size, tumor number, vascular invasion, BCLC stage, mode of operation (open or laparoscopic), operation duration, blood loss, blood transfusion or hepatic vascular occlusion during the operation.

**Table 2:** Relationship of perioperative CTC levels to patient characteristics

Characteristics	Postoperative vs. Preoperative CTC counts				
	Total (*N* = 139)	Decreased (*N* = 35)	No change (*N* = 46)	Increased (*N* = 58)	*P*
Age, years					0.153
≤ 50	80	25	25	30	
> 50	59	10	21	28	
Sex					0.577
Male	122	32	41	49	
Female	17	3	5	9	
HBsAg					0.521
Negative	21	7	5	9	
Positive	118	28	41	49	
Liver cirrhosis					0.077
No	36	13	7	16	
Yes	103	22	39	42	
Child-Pugh score					0.562*
A	132	32	44	56	
B	7	3	2	2	
Operation method					0.408
Open	123	33	39	51	
Laparoscopic	16	2	7	7	
Operation time (min)					0.247
≥ 240	68	21	19	28	
< 240	71	14	27	30	
Blood loss (ml)					0.313
> 200	67	19	18	20	
≤ 200	72	16	28	28	
Blood transfusion					0.318
Yes	28	10	7	11	
No	111	25	39	47	
Hepatic vascular occlusion					0.211
Yes	83	25	24	34	
No	56	10	22	24	
AFP, ng/mL					0.102
Negative (≤ 7.0)	39	5	16	18	
Positive (> 7.0)	100	30	30	40	
Largest tumor size, cm					0.067
≤ 5	61	10	25	26	
> 5	78	25	21	32	
No. of tumors					0.718
Single	106	26	37	43	
Multiple	33	9	9	15	
Macroscopic tumor thrombus					0.012
No	113	29	43	41	
Yes	26	6	3	17	
Vascular invasion					0.053
No	84	17	34	33	
Yes	55	18	12	25	
BCLC stage					0.089
0 + A	56	10	24	22	
B + C	83	25	22	36	

*Linear-by-linear association

### Prognostic significance of the surgery caused CTC count changes

**Fig. 2:**
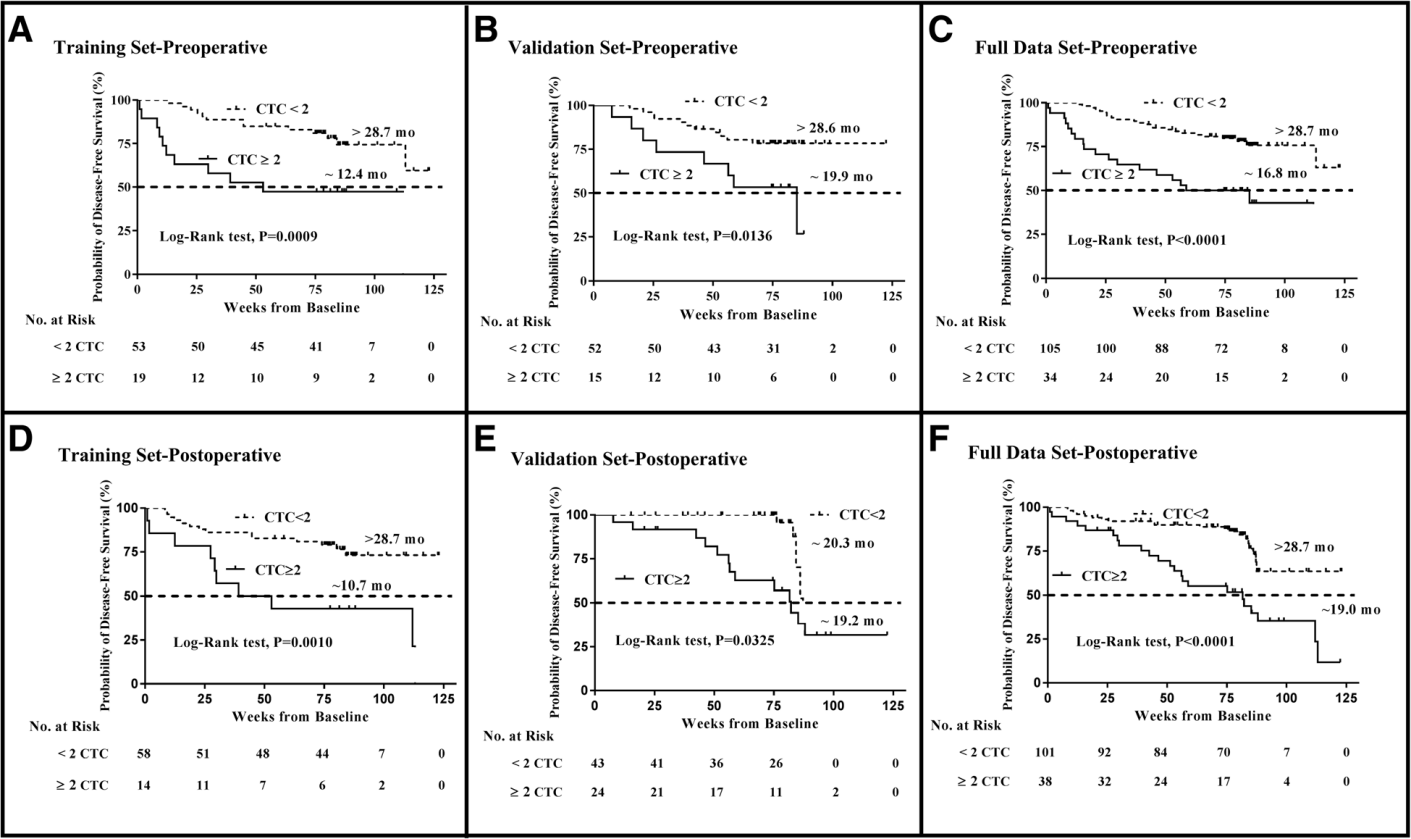
Kaplan-Meier estimates of DFS probabilities in patients with operable HCC using a cutoff value of 2 CTCs per 7.5 ml of peripheral blood. **a** Preoperative CTC < 2 or ≥ 2, training set; **b** Preoperative CTC < 2 or ≥ 2, validation set; **c** Preoperative CTC < 2 or ≥ 2, full data set; **d** Postoperative CTC < 2 or ≥ 2, training set; **e** Postoperative CTC < 2 or ≥ 2, validation set; **f** Postoperative CTC < 2 or ≥ 2, full data set

To investigate whether these perioperative CTC changes would have long-term effects on patients’ DFS and OS, the CTC level was selected that most clearly distinguished patients with longer DFS and OS from those with shorter ones. The 139 HCC patients in the cohort were randomly divided into two groups and analyzed, and their clinical characteristics and follow-up times did not significantly differ. The first group (training set, *n* = 72) was then used to select the CTC cutoff level. Thresholds of 1 to 10 cells for the perioperative levels were systematically correlated with DFS and OS. The results indicated that in 7.5 ml of blood, a threshold CTC value of 2 most significantly predicted patient outcome. This cutoff level was then validated using the second group (validation set, *n* = 67). For both DFS (Fig. 2) and OS (Additional file 2), the Kaplan-Meier estimates for all patient sets differed significantly (*P* < 0.05); thus, a cutoff level of 2 was used for further analyses.

**Fig. 3:**
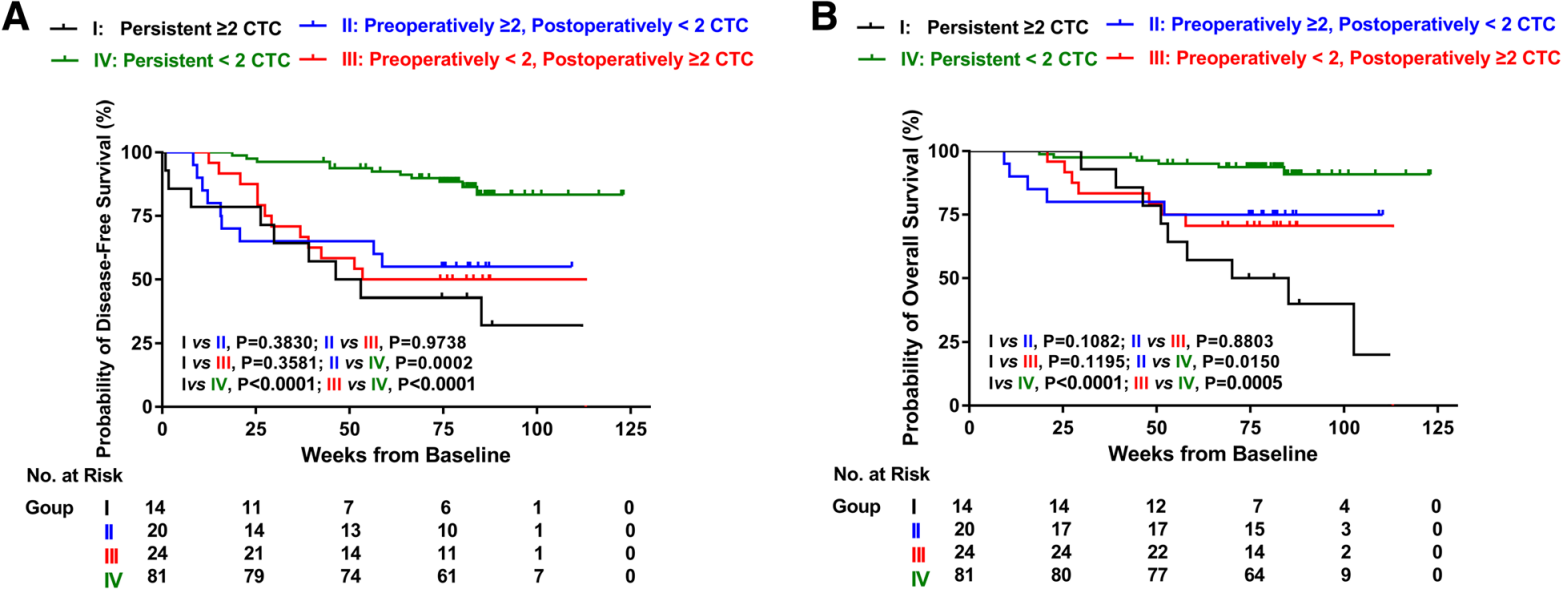
Kaplan-Meier estimates of DFS and OS probabilities in HCC patients with persistent CTC ≥ 2, change in CTCs from ≥2 to < 2, change in CTCs from < 2 to ≥2, and persistent CTC < 2 before and after surgery

Next, using a CTC of 2 as the cutoff value, 139 HCC patients were divided into four groups (Fig. 3): I, persistent levels of ≥2 CTC (*n* = 14); II, preoperatively ≥2 then postoperatively < 2 CTC (*n* = 20); III, preoperatively < 2 then postoperatively ≥2 CTC (*n* = 24); and IV, persistent levels of < 2 CTC (*n* = 81). The tendency between DFS and OS did not significantly differ. Patients in group I showed worse prognoses than group IV, with significantly shorter DFS (median survival, 11.6 months versus not reached; *P* < 0.0001) and OS (median survival, 18.1 months versus not reached; death, 71.4% versus 7.4%; *P* < 0.0001). Group I also had an increased risk of death compared with group II (median survival, 18.1 months versus not reached; death, 71.4% versus 25.0%; *P* = 0.1082) and group III (median survival, 18.1 months versus not reached; death, 71.4% versus 33.3%; *P* = 0.1195) in OS. Compared with group IV, patients in the other three groups had a significantly shorter DFS and OS (*P* < 0.05). Because patients in four groups showed significant differences in AFP, tumor size, tumor number, vascular invasion, macroscopic tumor thrombus and BCLC stage (Additional file 3), a multivariate Cox proportional regression analysis that included these factors was performed (to avoid potential bias, the BCLC stage was not included because it was associated with tumor characteristics and liver function). The results showed that this grouping was a strong independent predictor of DFS (HR, 0.620; 95% CI: 0.479–0.803; *P* = 0.000) and OS (HR, 0.608; 95% CI: 0.443–0.834; *P* = 0.002) (Table 3). Other tumor-related factors, including tumor size (DFS: HR, 4.840; 95% CI: 1.518–15.428; *P* = 0.008; OS: HR, 11.728; 95% CI: 1.448–94.962; *P* = 0.021) and macroscopic tumor thrombus (DFS: HR, 2.588; 95% CI: 1.174–5.706; *P* = 0.018; OS: HR, 2.795; 95% CI: 1.084–7.206; *P* = 0.033) remained significant and independent in the multivariate Cox regression. No other variables were included in the multivariate regression because they lacked significance in the univariate analysis.

**Table 3:** Univariate and multivariate Cox proportional regression analysis of factors associating with DFS and OS

Variables	Disease-free survival				Overall survival			
	Univariate analysis		Multivariate analysis		Univariate analysis		Multivariate analysis	
	HR (95% CI)	*P*	HR (95% CI)	*P*	HR (95% CI)	*P*	HR (95% CI)	*P*
Age, > 50 years vs. ≤ 50 years	0.739(0.398–1.373)	0.339			0.466 (0.207–1.054)	0.067		
Sex, male vs. female	0.505(0.156–1.637)	0.255			0.574 (0.136–2.431)	0.451		
HBsAg, positive vs. negative	2.528(0.780–8.192)	0.122			4.966 (0.673–36.617)	0.116		
Liver cirrhosis, yes vs. no	0.568(0.303–1.065)	0.078			0.799 (0.362–1.765)	0.580		
Child-Pugh score, B vs. A	1.398(0.430–4.543)	0.577			1.977 (0.590–6.624)	0.269		
No. of tumors, multiple vs. single	2.287(1.209–4.327)	0.011	0.939(0.475–1.855)	0.856	3.223(1.505–6.902)	0.003	1.379 (0.618–3.078)	0.432
Tumor size, ≤ 5 cm vs. > 5 cm	10.403(3.710–29.173)	0.000	4.840(1.518–15.428)	0.008	27.058 (3.679–199.020)	0.001	11.728 (1.448–94.962)	0.021
Macroscopic tumor thrombus, yes vs. no	6.836(3.567–13.100)	0.000	2.588(1.174–5.706)	0.018	8.194 (3.646–18.413)	0.000	2.795 (1.084–7.206)	0.033
Vascular invasion, yes vs. no	6.145(3.124–12.085)	0.000	1.816(0.766–4.307)	0.176	5.933 (2.584–13.622)	0.000	1.491 (0.510–4.357))	0.466
AFP, positive vs. negative	2.349(1.043–5.288)	0.039	1.172(0.474–2.899)	0.731	2.781 (0.966–8.001)	0.058	1.709 (0.548–5.332)	0.356
BCLC stage, B + C vs. 0 + A	8.695(3.103–24.370)	0.000			11.212 (2.663–47.204)	0.001		
Group	0.529(0.414–0.676)	0.000	0.620(0.479–0.803)	0.000	0.484 (0.357–0.656)	0.000	0.608 (0.443–0.834)	0.002

## Discussion

Surgical liver resection is the most effective therapy for early-stage HCC patients [14]. However, of the HCC patients undergoing surgery for resectable disease, more than 50% will develop subsequent metastases [3]. The number of CTCs that the CellSearch™ System detects in the vasculature has been shown to correlate with HCC patient survival and prognosis [8, 15]. However, using this technology for HCC is under debate as its CTC detection rate appears to associate with EpCAM expression in individual tumors [16]. EpCAM could serve as a biomarker for tumor-initiating cells in HCC [17], because EpCAM-positive CTCs are considered a subtype of circulating cancer stem cells with stronger metastatic potential. But only approximately 35% of HCC cases express EpCAM [18]; thus, detection sensitivity would be low and would include many false negative results. In this study, the detection ratios (≥ 1 CTC) before and after surgery were 43.9% and 54.0%, respectively, which is consistent with previous reports and the EpCAM expression pattern in HCC [8, 15, 19–22].

Many studies have shown that tumor biopsy and resection can lead to tumor cell dissemination [23, 24]. However, the impact of the increased CTCs remains controversial [9]. In our study, we found a propensity for increasing both the incidence of CTC detection and mean CTC counts postoperatively (54%, mean 1.54 cells) versus preoperatively (43%, mean 1.13 cells). The postoperative CTC counts increased in 41.7% of patients, decreased in 25.2% of patients and did not change in 33.1% of patients. The postoperative CTC counts changed (either increased or decreased) in 66.9% of HCC patients, indicating that surgery caused the CTC changes. The association between the change in perioperative CTC counts and clinical parameters was analyzed next. We found that the increase in postoperative CTC counts was significantly associated with the macroscopic tumor thrombus condition, suggesting that carefully handling macroscopic tumor thrombi during the operation may reduce the number of CTCs released, thus improving patient outcomes.

Some evidence showed that HCC tended to spread from the portal system in the early stage and was driven into the blood stream from the hepatic vein tumor thrombus when moving and rotating the liver [25–27]. A “no-touch” technique might prevent the spread of cancer cells to vein during liver resection, which could reduce CTC dissemination [28]. Ligating inflow and outflow vessels without hilus dissection before manipulating the tumor could completely block hepatic blood flow on the diseased side [29, 30]. In our study, 5 patients with HCC used this technique, and none of them showed elevated CTCs postoperatively. Hence, operative modifications may reduce the occurrence of postoperative CTC increases, but studies of more patients with longer survival times are needed to confirm this.

Moreover, our data indicated that increased or decreased postoperative CTC counts were not significantly associated with patients’ DFS or OS (data not shown), as both preoperative and postoperative CTC counts indicated patients’ prognoses. We used a CTC count of 2 as the cutoff value. Patients with increased postoperative CTC counts (from preoperative CTC < 2 to postoperative CTC ≥ 2) had significantly shorter DFS and OS than did patients with persistent CTC < 2. Patients with persistent levels of ≥2 CTC before and after surgery had the worst prognoses, while those with persistent levels of < 2 CTC had the longest DFS and OS.

## Conclusions

In conclusion, our data demonstrated the effect of surgical liver resection on CTCs in patients with HCC. Our findings supported the common occurrence of postoperative CTC increases but also indicated that this event may be prevented by operative modifications. These observations also suggested that detecting perioperative CTCs may be a strong indicator of the response to the HCC curative resection and therapeutic approach, which directly targets CTCs and could hold great promise as a perioperative adjuvant treatment.

## Additional files

Additional file 1: Results of CTC detection at different time-points in 12 HCC patients undergoing curative liver resection. (DOCX 957 kb)

Additional file 2: Kaplan-Meier estimates of OS probabilities in patients with operable HCC using a cutoff value of 2 CTCs per 7.5 ml of peripheral blood. (**A**) Preoperative CTC < 2 or ≥ 2, training set; (**B**) Preoperative CTC < 2 or ≥ 2, validation set; (**C**) Preoperative CTC < 2 or ≥ 2, full data set; (**D**) Postoperative CTC < 2 or ≥ 2, training set; (**E**) Postoperative CTC < 2 or ≥ 2, validation set; (**F**) Postoperative CTC < 2 or ≥ 2, full data set. (TIF 1682 kb)

Additional file 3: Baseline characteristics of HCC patients in four groups. (DOCX 1812 kb)

## Abbreviations

AFP: α-fetoprotein
BCLC: Barcelona Clinic Liver Cancer
CTCs: circulating tumor cells
DFS: disease-free survival
HBsAg: hepatitis B surface antigen
HBV: hepatitis B virus
HCC: hepatocellular carcinoma
HCV: hepatitis C virus
OS: overall survival
