# Supplementary material for: Effect of surgical liver resection on circulating tumor cells in patients with hepatocellular carcinoma
Source: BMC Cancer. 2018 Aug 20;18:835. doi: 10.1186/s12885-018-4744-4 (PMC6102841; doi:10.1186/s12885-018-4744-4)
Supplement: Supplementary file 1 — Results of CTC detection at different time-points in 12 HCC patients undergoing curative liver resection. (DOCX 957 kb) [file 12885_2018_4744_MOESM1_ESM.docx]

**Additional file 1. Results of CTC detection at different time-points in 12 HCC patients undergoing curative liver resection.**

| **Case** | **No. of CTCs in 7.5 mL of blood** | | | |
| --- | --- | --- | --- | --- |
|  | **Preoperative** | **Immediately after surgery** | **3-days after**  **surgery** | **7-days after**  **surgery** |
| 1 | 0 | 0 | 0 | 0 |
| 2 | 0 | 0 | 0 | 0 |
| 3 | 0 | 0 | 0 | 1 |
| 4 | 1 | 1 | 0 | 0 |
| 5 | 0 | 0 | 0 | 0 |
| 6 | 0 | 1 | 0 | 0 |
| 7 | 0 | 0 | 1 | 1 |
| 8 | 1 | 3 | 0 | 0 |
| 9 | 1 | 0 | 0 | 0 |
| 10 | 5 | 3 | 3 | 3 |
| 11 | 2 | 0 | 1 | 0 |
| 12 | 8 | 4 | 4 | 3 |
