# Supplementary figures and images for: Effect of surgical liver resection on circulating tumor cells in patients with hepatocellular carcinoma
Source: BMC Cancer. 2018 Aug 20;18:835. doi: 10.1186/s12885-018-4744-4 (PMC6102841; doi:10.1186/s12885-018-4744-4)

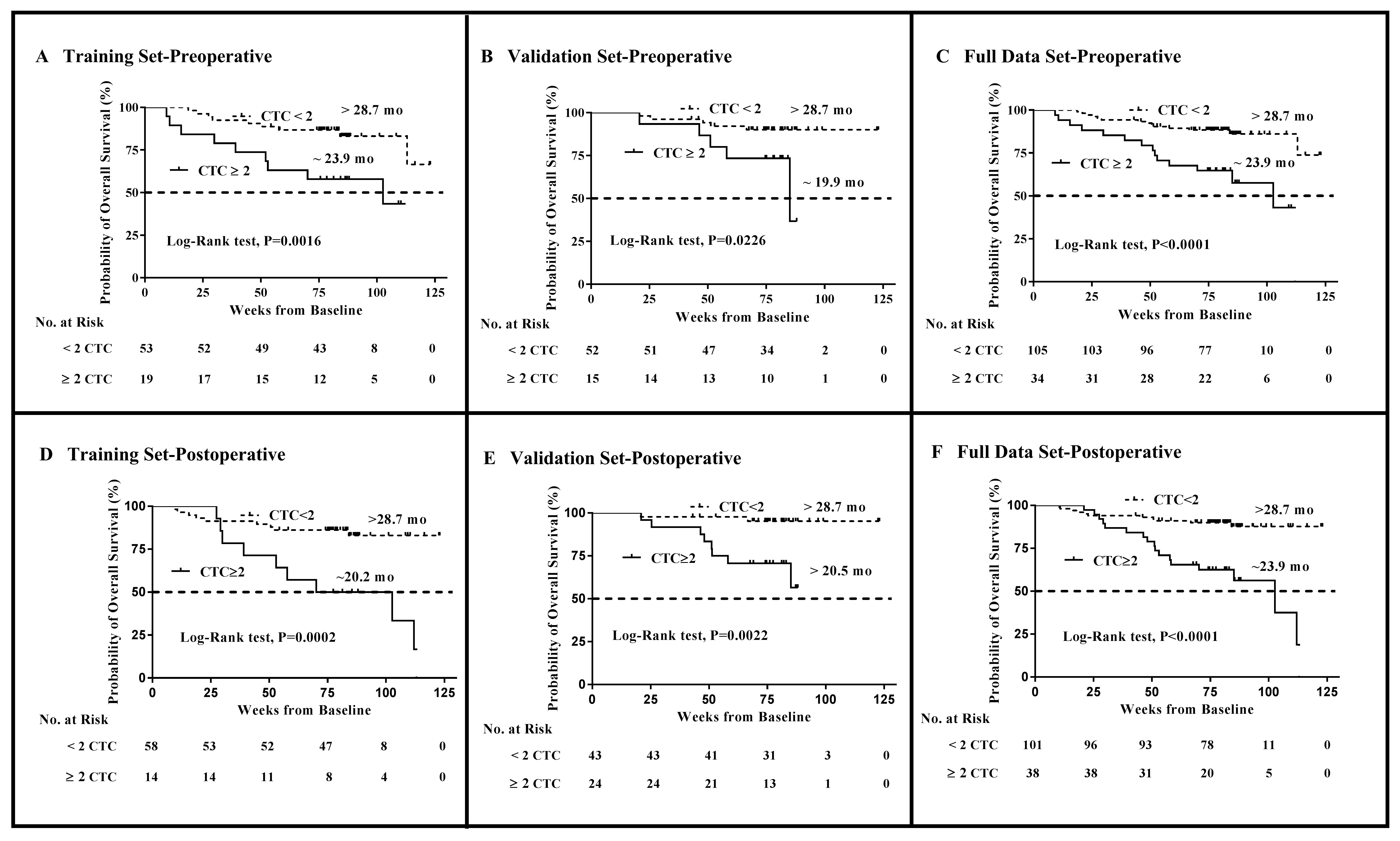

Supplement: Supplementary file 2 — Kaplan-Meier estimates of OS probabilities in patients with operable HCC using a cutoff value of 2 CTCs per 7.5 ml of peripheral blood. (A) Preoperative CTC < 2 or ≥ 2, training set; (B) Preoperative CTC < 2 or ≥ 2, validation set; (C) Preoperative CTC < 2 or ≥ 2, full data set; (D) Postoperative CTC < 2 or ≥ 2, training set; (E) Postoperative CTC < 2 or ≥ 2, validation set; (F) Postoperative CTC < 2 or ≥ 2, full data set. (TIF 1682 kb) [file 12885_2018_4744_MOESM2_ESM.tif]
