# Supplementary material for: Effect of surgical liver resection on circulating tumor cells in patients with hepatocellular carcinoma
Source: BMC Cancer. 2018 Aug 20;18:835. doi: 10.1186/s12885-018-4744-4 (PMC6102841; doi:10.1186/s12885-018-4744-4)
Supplement: Supplementary file 3 — Baseline characteristics of HCC patients in four groups. (DOCX 1812 kb) [file 12885_2018_4744_MOESM3_ESM.docx]

**Additional file 3. Baseline characteristics of HCC patients in four groups.**

| **Clinical characteristics** | **No. of patients** | | | | | | | | **P** |
| --- | --- | --- | --- | --- | --- | --- | --- | --- | --- |
|  | **Total (N = 139)** | | **Group I (N = 14)** | | **Group II (N = 20)** | | **Group III (N = 24)** | **Group IV (N = 81)** |  |
| Age, years |  |  | |  | |  | |  | 0.382 |
| ≤ 50 | 80 | 7 | | 15 | | 13 | | 45 |  |
| > 50 | 59 | 7 | | 5 | | 11 | | 36 |  |
| Sex |  |  | |  | |  | |  | 0.625* |
| Male | 122 | 12 | | 19 | | 21 | | 70 |  |
| Female | 17 | 2 | | 1 | | 3 | | 11 |  |
| HBsAg |  |  | |  | |  | |  | 0.360* |
| Negative | 21 | 2 | | 2 | | 2 | | 15 |  |
| Positive | 118 | 12 | | 18 | | 22 | | 66 |  |
| Liver cirrhosis |  |  | |  | |  | |  | 0.715 |
| No | 36 | 4 | | 6 | | 4 | | 22 |  |
| Yes | 103 | 10 | | 14 | | 20 | | 59 |  |
| Child-Pugh score |  |  | |  | |  | |  | 0.172* |
| A | 132 | 13 | | 18 | | 22 | | 79 |  |
| B | 7 | 1 | | 2 | | 2 | | 2 |  |
| AFP, ng/mL |  |  | |  | |  | |  | **0.044** |
| Negative | 39 | 3 | | 1 | | 6 | | 29 |  |
| Positive | 100 | 11 | | 19 | | 18 | | 52 |  |
| Tumor size, cm |  |  | |  | |  | |  | **0.014** |
| ≤ 5 | 61 | 4 | | 3 | | 12 | | 42 |  |
| > 5 | 78 | 10 | | 17 | | 12 | | 39 |  |
| No. of tumors |  |  | |  | |  | |  | **0.014*** |
| Single | 106 | 8 | | 13 | | 18 | | 67 |  |
| Multiple | 33 | 6 | | 7 | | 6 | | 14 |  |
| Vascular invasion  No  Yes  Macroscopic tumor thrombus | 84  55 | 6  8 | | 7  13 | | 13  11 | | 58  23 | **0.008**  **0.011*** |
| No | 113 | 8 | | 17 | | 16 | | 72 |  |
| Yes | 26 | 6 | | 3 | | 8 | | 9 |  |
| BCLC stage |  |  | |  | |  | |  | **0.017** |
| 0 + A | 56 | 3 | | 3 | | 10 | | 40 |  |
| B + C | 83 | 11 | | 17 | | 14 | | 41 |  |

*Linear-by-linear association
